# Long-Term Treatment of Gaucher Disease with Velaglucerase Alfa in ERT-Naïve Patients from the Gaucher Outcome Survey (GOS) Registry

**DOI:** 10.3390/jcm13102782

**Published:** 2024-05-09

**Authors:** Patrick Deegan, Heather Lau, Deborah Elstein, Diego Fernandez-Sasso, Pilar Giraldo, Derralynn Hughes, Ari Zimran, Majdolen Istaiti, Noga Gadir, Jaco Botha, Shoshana Revel-Vilk

**Affiliations:** 1 Lysosomal Disorders Unit, Cambridge University Hospitals, Hills Road, Cambridge CB2 0QQ, UK; 2 Langone Medical Center, New York University, 333 E 33rd St, New York, NY 10016, USA; heather.lau4@gmail.com; 3 Takeda Pharmaceuticals International AG, Thurgauerstrasse 130, 8152 Zurich, Switzerland; debby.elstein@gmail.com (D.E.); noga.gadir-david@takeda.com (N.G.); jaco.botha@takeda.com (J.B.); 4 Instituto William Osler, Paraguay 2935, Buenos Aires C1425 BRI, Argentina; diegofernandezsasso@criocenter.com; 5 En el Centro de Investigación Biomédica en Red (CIBER) de Enfermedades Raras, IIS Aragon, C. de San Juan Bosco 13, 50009 Zaragoza, Spain; giraldocastellano@gmail.com; 6 Translational Research Unit, IIS Aragon, Paseo de Isabel la Católica 1-3, 50009 Zaragoza, Spain; 7 Lysosomal Storage Disorders Unit, Department of Haematology, Royal Free Hospital, UCL Medical School, Pond Street, London NW3 2QG, UK; derralynnhughes@nhs.net; 8 Gaucher Unit, Shaare Zedek Medical Center, Shmuel (Hans) Beyth St 12, Jerusalem 9103102, Israel; azimran@gmail.com (A.Z.); joleenist@szmc.org.il (M.I.); srevelvilk@gmail.com (S.R.-V.); 9 The Faculty of Medicine, Hebrew University, Campus Ein Kerem, Jerusalem 9112102, Israel

**Keywords:** velaglucerase alfa, enzyme replacement therapy, ERT, Gaucher disease, GOS, registry, long term

## Abstract

**Background**: Gaucher disease (GD) is a rare, autosomal, recessive condition characterized by hepatosplenomegaly, thrombocytopenia, anemia, and bone abnormalities, often requiring life-long treatment. Velaglucerase alfa has improved hematologic and visceral parameters in clinical trials; however, limited long-term efficacy and safety data are available. **Methods**: The Gaucher Outcome Survey (GOS), a structured and validated international registry for patients with confirmed GD, provides an opportunity to evaluate long-term data from patients receiving velaglucerase alfa. **Results**: This analysis included 376 treatment-naïve children and adults with GD enrolled in GOS, including 20 with type 3 GD, who initiated velaglucerase alfa through participation in clinical trials or as part of their clinical management and continued treatment for a mean (range) time of 6.6 (0.003–18.6) years. Initial improvements in hematologic and visceral parameters and the biomarkers glucosylsphingosine (lyso-GL1) and chitotriosidase were observed after one year of treatment and were maintained throughout the follow-up period. Of 129 (34.3%) patients who developed adverse events during the follow-up period, events were considered related to treatment in 33 (8.8%). None led to treatment discontinuation. There were 21 deaths overall, none of which were considered related to treatment. **Conclusions**: This analysis of data from the GOS registry supports the safety and efficacy of velaglucerase alfa in patients with GD.

## 1. Introduction

Gaucher disease (GD) is a rare, autosomal, recessive inherited disorder caused by mutations of the *GBA1* gene. This causes deficiency of the lysosomal enzyme β-glucocerebrosidase and subsequent accumulation of glucocerebroside in cells of the monocyte-macrophage system [1]. Type 1 GD is characterized by splenomegaly, hepatomegaly, thrombocytopenia, anemia, and bone abnormalities, including radiographic changes and pain, while types 2 and 3 are also associated with specific neurological manifestations [2].

Before the availability of effective pharmacological agents for GD, splenectomy was the preferred treatment, conducted to mitigate life-threatening thrombocytopenia. However, this practice is not without risk, including deterioration in bone and liver involvement and an association with long-term vulnerability to infections. In developed healthcare economies, it is now reserved for the resistant or complicated cases of splenomegaly. Since 1991, six pharmacological therapies have been approved for the treatment of patients with GD. The first, the enzyme replacement therapy (ERT) alglucerase (Genzyme Corporation), was approved for use in 1991 and was subsequently replaced by the recombinant imiglucerase (Genzyme Corporation), which was approved in 1994. This was followed by a further two ERTs (velaglucerase alfa [Shire, now Takeda] and taliglucerase alfa [Pfizer and Protalix BioTherapeutics]), as well as two substrate reduction therapies (SRTs; miglustat [Actelion, now Johnson & Johnson] and eliglustat [Sanofi]). Clinical trial outcomes have demonstrated that these agents can result in improvements in the systemic manifestations of GD, including those affecting patients with type 3 GD [3], and they are now the mainstay of therapy in non-neuronopathic GD [4,5,6,7,8,9,10].

As with many chronic disorders, patients with GD often require life-long treatment. However, limited long-term data are available for most ERTs and SRTs. Treatment of up to 20 years with imiglucerase has been evaluated using data from the International Collaborative Gaucher Group (ICGG) registry [11], while clinical data for up to eight years are available for the oral SRT, eliglustat [12]. Published long-term data for velaglucerase alfa extends to a maximum of seven years’ treatment duration using data from clinical trials [10], while over 12 years of outcome data have been captured in the Gaucher Outcomes Survey (GOS), initiated in 2010 [13]. Moreover, many patients who took part in the velaglucerase alfa clinical trial program subsequently enrolled in GOS, resulting in a pool of patients with long-term data beyond the commercial availability of the drug, up to a maximum of ~18 years. The aim of these analyses was to evaluate the long-term efficacy and safety of velaglucerase alfa in patients with GD, using data from velaglucerase alfa clinical trials and the GOS registry.

## 2. Materials and Methods

### 2.1. Study Design

GOS is an ongoing, international, observational, long-term disease-specific registry established in 2010 for patients with a confirmed GD diagnosis, regardless of their GD type or treatment status (NCT03291223). The registry collects data from routine clinical practice and provides validated information on patient characteristics, clinical outcomes, and more. The objectives of GOS include: to evaluate the long-term safety and effectiveness of velaglucerase alfa; to characterize patients receiving velaglucerase alfa or other GD-specific treatments; to gain a better understanding of the natural history of GD; and to serve as a database for evidence-based management of GD over time in routine clinical practice.

### 2.2. Patients

GOS is open to patients of any age or gender with a confirmed GD diagnosis, regardless of their GD type or treatment status, who are receiving treatment at participating centers [13,14,15]. To be enrolled in GOS, patients are required to have a confirmed diagnosis of GD demonstrated biochemically by reduced glucocerebrosidase activity or genetically by *GBA1* genotyping, and to provide written informed consent. For patients <18 years of age (<16 years of age in the UK), consent is obtained from a parent or legal representative, along with assent where appropriate. Patients participating in clinical trials are excluded from participation in GOS for the duration of their involvement, but may (re-)enroll after completion of the trial.

For inclusion in this analysis, patients were required to have confirmed type 1 or type 3 GD, to have participated in velaglucerase alfa clinical trials and/or be enrolled in GOS, be treatment-naïve prior to velaglucerase alfa initiation, and to have received only velaglucerase alfa thereafter. Patients with type 2 GD, a rapidly neurodegenerative disease with no approved drug therapies, were excluded from the analysis. Additionally, patients who had a record of treatment with any other GD-specific agent at any time were excluded from the analysis. Patients who had participated in clinical trials for GD prior to GOS enrollment were considered treatment-naïve if they had not received GD-specific therapy for at least 30 months prior to clinical trial enrollment.

### 2.3. Data Collection

For patients who enrolled into GOS following participation in clinical trials, available data from both the clinical trial database and the GOS database were evaluated. GOS data were collected via web-based electronic case report forms (eCRF) and included patient demographics, diagnosis, physical characteristics, treatment type and dosing information, hematologic and visceral parameters, adverse events (AEs), plasma/serum chitotriosidase activity, and plasma/serum or dried blood spot (DBS) analysis of glucosylsphingosine (lyso-GL1) concentrations. Liver and spleen volumes are measured by quantitative abdominal magnetic resonance imaging (MRI), computed tomography (CT), or 3D ultrasound (using a conversion algorithm as described previously [16]) at approximately annual intervals. Orthopedic imaging modalities included plain radiographs (X-rays), ultrasound, MRI, and CT.

All patient data are handled in accordance with relevant global and local regulations and best practice and with Good Pharmacoepidemiological Practice, Good Research for Comparative Effectiveness principles, and the principles of the International Conference on Harmonization Good Clinical Practice guidelines.

### 2.4. Analyses

Assessments included changes in hemoglobin concentration from baseline (defined as the first dose of velaglucerase alfa) to last available time-point, platelet count, spleen and liver volume, plasma chitotriosidase, and blood or plasma lyso-GL1 concentrations for the overall population, as well as for a subgroup of patients diagnosed with type 3 GD. Liver and spleen volumes were expressed as multiples of normal (MN), calculated as a percentage of the patient’s body weight in kg divided by normal liver or spleen volume, defined as 2.5 mL/kg and 0.2 mL/kg, respectively. The proportion of patients meeting (or maintaining) diagnostic thresholds for anemia, thrombocytopenia, hepatomegaly, and splenomegaly were reported using accepted reference values [17,18]. Additionally, the proportion of patients meeting accepted therapeutic goals for patients with GD were reported [17,19]. Reference thresholds and treatment goals are summarized in Table 1. Patients with missing treatment information were excluded from the analysis.

### 2.5. Statistical Analysis

Mean annual rates of change in hematologic and visceral outcomes and biomarker concentrations were calculated using mixed-effects linear regression analysis for patients with ≥3 measurements recorded in GOS. Summary statistics were calculated for subgroups of patients with available data at baseline and at each respective time-point. Outlying values were investigated and, if deemed improbable, were excluded from analyses. Percentages were calculated using the number of patients with available data as the denominator. Statistical analyses were performed using SAS^®^ software version 9.4. No between-group statistical comparisons were performed.

## 3. Results

### 3.1. Patient Demographics and Clinical Characteristics

As of February 25, 2023, 2074 patients with type 1 or type 3 GD were enrolled in GOS. Of these, 376 patients from 13 countries (Argentina, Austria, Canada, France, Germany, the UK, Israel, Italy, Paraguay, Poland, Russia, Spain, and the USA) received only velaglucerase alfa up to the data extract date and were included in the primary cohort for this analysis. Patients initiated velaglucerase alfa treatment either prior to enrollment in GOS (as part of routine clinical care or participation in velaglucerase alfa clinical trials), or directly upon enrollment into GOS, and remained on velaglucerase alfa for a mean (SD) of 6.6 (4.3) years (*n* = 359). Overall, 215 (57.2%) patients were female, 102 (27.1%) were <18 years of age (range: 0.1–16.4 years) at velaglucerase alfa initiation, and 274 (72.9%) were ≥18 years (Table 2). Thirty-two (8.5%) had undergone a total splenectomy, 20 (62.5%) of whom were female. For 25 patients with recorded dates of splenectomy, the mean (range) time between splenectomy and velaglucerase alfa initiation was 26.2 (1.7–50.0) years. Based on investigator assessment, the majority of patients (337; 94.4%) had a diagnosis of type 1 GD, and 20 patients (5.6%) had a diagnosis of type 3 GD. Overall, the mean age at symptom onset was 19.1 years, the mean age at diagnosis was 22.0 years, and the mean age at treatment initiation was 33.3 years. Genotype information was available for 256 patients overall; 129 (50.4%) were N370S (c.1226A > G; p.Asp409Ser; N409S current nomenclature) homozygotes, 89 (34.8%) were N370S heterozygotes, 12 (4.7%) were L444P (c.1448T > C; p.Leu483Pro; L483P current nomenclature) heterozygous, and 7 (2.7%) were L444P homozygous (Table 2). Patient demographics of the primary cohort were similar to those for 1698 patients with type 1 or type 3 GD enrolled in GOS who had received treatment other than or as well as velaglucerase alfa or had not received any treatment, and who were not evaluated further in this analysis.

The subgroup of 20 patients with a clinical diagnosis of type 3 GD was included in a secondary analysis cohort. Of these, 16 were female and none had undergone a total splenectomy. Seven were from the US, eight from Israel, and five from the UK. Patients with type 3 GD had a mean age at symptom onset of 3.6 years, mean age at diagnosis of 4.0 years, and mean age at treatment initiation of 7.4 years. Sixteen patients with type 3 GD had genotype information recorded in GOS; 12 were confirmed as having L444P-containing genotypes (seven homozygous and five heterozygous; Table 2) and four had no specific genotype recorded. The mean (SD) duration of treatment was 4.6 (3.9) years for patients with type 3 GD.

### 3.2. Velaglucerase Alfa Dose

Dosing information was available for 359 (95.5%) patients. The mean (SD) starting dose of velaglucerase alfa was 45.7 (21.5) U/kg for the overall cohort (*n* = 359): 42.3 (19.9) U/kg for adults (*n* = 261) and 54.6 (23.1) U/kg for children (*n* = 98). Approximately half (176; 49.0%) received velaglucerase alfa at doses of 45–≤60 U/kg, and 345 (96.1%) were dosed every other week. Mean starting doses were lowest in Israel, Poland, Russia, and Spain (all <40 U/kg) and highest in Argentina, France, Italy, Paraguay, the UK, and the USA (all >50 U/kg). There were no clear associations between starting doses and dosing instructions from clinical trials in each respective country, which were standard and mandated by the trial protocol. Doses remained consistent over time for most patients, with an overall mean (SD) last available dose of 43.5 (19.2) U/kg. Patients from Canada, Paraguay, and Russia reported dose reductions over time (from 46.9 to 39.2 U/kg, 54.5 to 47.4 U/kg, and 37.8 to 32.1 U/kg, respectively), while doses in all other countries remained stable.

Patients with type 3 GD (*n* = 20) had a higher mean (SD) starting dose of 63.7 (20.8) U/kg and a mean (SD) last available dose of 64.1 (18.7) U/kg. Thirteen (65.0%) patients received doses within the 45–60 U/kg range, and 18 (90.0%) received treatment every other week.

### 3.3. Hemoglobin Concentration

Substantial improvements in hemoglobin concentrations were observed with velaglucerase alfa treatment for 309 (82.2%) patients with ≥1 hemoglobin assessment at any time. These improvements were maintained long-term for up to 19 years of follow-up (overall mean [SE] estimated annual rate of change, 0.05 [0.01] g/L/year; Table 3). For patients with a baseline and ≥1 post-baseline value, mean (SD) hemoglobin concentrations increased from 118.6 (18.5) g/L at baseline to 131.8 (16.3) g/L after one year of treatment (*n* = 138) and were maintained long term, reaching a mean (SD) of 135.5 (14.1) g/L for 15 patients with data at 15 years. Similar increases were achieved by patients across all age/sex categories in line with age-specific thresholds (≥112 g/L to ≥127 g/L, summarized in Table 1 [17,20]), although with considerable variation at time-points after 6 years of treatment owing to small patient numbers.

**Figure 1:**
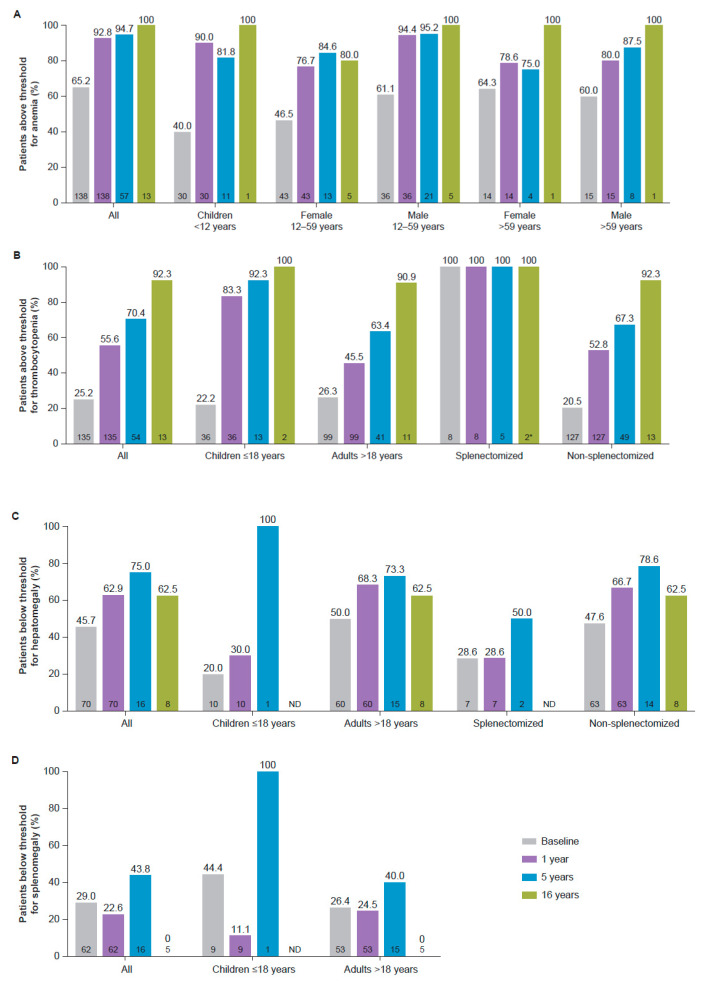
Proportion of patients above standard diagnostic thresholds for (**A**) anemia and (**B**) thrombocytopenia, and below thresholds for (**C**) hepatomegaly and (**D**) splenomegaly. * For thrombocytopenia, the last time-point in splenectomized patients was 15 years. Thresholds for anemia, thrombocytopenia, hepatomegaly, and splenomegaly are summarized in Table 1. Sample size at each time-point is indicated within each bar (bottom). ND, no data.

Overall, 128/138 (92.8%) patients achieved hemoglobin concentrations above age- and sex-relevant diagnostic thresholds for anemia after one year, increased from 90 (65.2%) at baseline, with similar responses achieved across all age/sex categories (Figure 1A). GD therapeutic targets for hemoglobin concentrations considered to represent normalization to the general population for age and sex (Table 1 [17,19]) were achieved by 96.2% of the patients overall after one year.

For patients with type 3 GD, small increases in hemoglobin concentrations were observed with long-term velaglucerase alfa treatment, from mean (SD) 103.0 g/L at baseline to 118.9 g/L at one year (*n* = 10), maintained up to 14 years, and a mean (SE) overall estimated annual rate of change of 0.13 (0.05) g/L over 11 years (*n* = 17; Table 3).

### 3.4. Platelet Counts

Substantial improvements in platelet counts were observed with velaglucerase alfa treatment for 311 (82.7%) patients with ≥1 platelet count assessment at any time. Improvements were maintained long-term up to 19 years (overall mean [SE] estimated annual rate of change, 0.35 [0.04] × 10^9^/L; Table 3). For patients with a baseline and ≥1 post-baseline value, mean (SD) platelet counts increased from 115.7 (72.9) × 10^9^/L at baseline to 178.8 (91.0) × 10^9^/L after one year (*n* = 135) and were maintained long-term, reaching a mean (SD) of 191.9 (41.5) × 10^9^/L in 15 patients with data at 15 years. Children ≤18 years of age had higher baseline platelet counts and greater increases after one year of treatment than adults (128.8 to 225.4 × 10^9^/L, *n* = 36 vs. 111.0 to 161.8 × 10^9^/L, *n* = 99, respectively). Patients who had undergone total splenectomy had higher baseline platelet counts and greater absolute increases to one year than non-splenectomized patients (233.3 to 321.5 × 10^9^/L, *n* = 8 vs. 108.3 to 169.8 × 10^9^/L, *n* = 127, respectively), maintained with long-term treatment.

Platelet counts above the WHO-established threshold for thrombocytopenia of ≥150 × 10^9^/L [18] were achieved by 75 (55.6%) patients by one year (83.3% of children and 45.5% of adults), compared with 34 (25.2%) at baseline (22.2% children and 26.3% adults; Figure 1B). Therapeutic targets for GD (1.5- to 2-fold increase by 1 year, summarized in Table 1 [17,19]) were achieved by 95 (70.4%) patients after one year.

For patients with type 3 GD, initial increases in platelet counts were observed with long-term velaglucerase alfa treatment, from a mean (SD) of 143.6 (82.8) at baseline to 239.4 (92.6) at one year (*n* = 10), remaining relatively stable thereafter. The mean (SE) estimated annual rate of change was −0.31 (0.39) × 10^9^/L (*n* = 18; Table 3).

### 3.5. Liver Volume

For 178 (47.3%) patients with ≥1 liver volume assessment at any time, patients with baseline liver volumes >1.5 MN (after adjustment for body weight) showed greater decreases over time compared with patients with near-normal baseline liver volumes (≤1.5 MN); near-normal liver volumes, once achieved, were maintained for up to 16 years. The overall mean (SE) estimated annual rate of change was −0.001 (0.0003) MN (Table 3).

Mean (SD) liver volume decreased from 2.2 (1.57) MN at baseline to 1.2 (0.32) MN at one year (*n* = 70); decreases were maintained long-term, reaching a mean (SD) of 1.2 (0.14) MN at 16 years for eight patients with available data. Children had larger liver volumes at baseline compared with adults (2.7 MN vs. 2.1 MN, respectively), but achieved similar liver volumes after one year of treatment (1.3 vs. 1.2 MN, respectively), and this was maintained long-term. Similarly, splenectomized patients (*n* = 7) had larger liver volumes than non-splenectomized patients (*n* = 63) at baseline (3.0 vs. 2.1 MN, respectively), but achieved similar liver volumes after one year (1.3 MN vs. 1.2 MN, respectively).

The proportion of patients with no hepatomegaly (≤1.25 MN, defined in Table 1 [11,21,22]) increased from 32 (45.7%) at baseline to 44 (62.9%) at one year and remained at ≥60.0% over 16 years (Figure 1C). GD therapeutic targets considered to represent normalization of liver volume (<1.5 MN, defined in Table 1 [17,19]) were achieved by 69 (98.6%) patients after one year of treatment, compared with 41 (58.6%) patients at baseline.

For patients with type 3 GD, liver volume assessments were unchanged with long-term velaglucerase alfa treatment (mean [SE] estimated annual rate of change −0.005 [0.003] MN, *n* = 9; Table 3).

### 3.6. Spleen Volume

Overall, 188 patients had ≥1 assessment of spleen volume during the follow-up period. Patients with spleen volumes >10 MN (after adjustment for body weight) showed rapid initial decreases in spleen volume, compared with little change in most patients with baseline spleen volumes ≤10 MN. Spleen sizes of <10 MN, once achieved, were maintained or further reduced with long-term treatment up to 16 years. The overall mean (SE) estimated annual rate of change was −0.03 (0.00) MN (Table 3).

A total of 62 patients had a baseline and ≥1 post-baseline assessment. For this cohort, mean (SD) spleen volumes remained stable throughout the follow-up period, from a baseline of 7.2 (4.41) MN (*n* = 62) to 6.2 (0.76) MN (*n* = 5) after 16 years. Although 47 (75.8%) patients with available data achieved the therapeutic target for GD of <8.0 MN (defined in Table 1 [17,19]) at one year, the proportion of patients with spleen volume ≤5.0 MN, the standard diagnostic threshold for splenomegaly [17], did not exceed 50% (Figure 1D).

The number of patients with type 3 GD with spleen volume measurements was insufficient for the calculation of the estimated annual rate of change over time.

### 3.7. Change in Clinical Biomarkers over Time

Overall, 210 patients had ≥1 plasma chitotriosidase assessment and 191 patients had ≥1 blood or plasma lyso-GL1 assessment during the follow-up period. Substantial decreases in both biomarkers were observed within one year of treatment initiation, with reductions maintained long-term for up to 16 years for both chitotriosidase and lyso-GL1. Overall mean (SE) estimates for annual rate of change were −135.4 (38,403) nmol/mL/h for chitotriosidase and −1.08 (0.20) ng/mL for lyso-GL1 (Table 3).

Change from baseline was assessed for 68 patients with both a baseline and ≥1 post-baseline measurement of chitotriosidase and for 66 patients with a baseline and ≥1 post-baseline measurement of lyso-GL1. Chitotriosidase concentrations decreased from 11,590.0 nmol/mL/h at baseline to 3138.4 nmol/mL/h at one year, with further reductions observed over the follow-up period, reaching 2062.7 nmol/mL/h for six patients after 10 years. Lyso-GL1 concentrations decreased from 258.1 ng/mL at baseline to 110.9 ng/mL at one year, with further reductions over time, reaching 42.7 ng/mL for five patients after 10 years.

Baseline lyso-GL1 concentrations were lower for individuals whose samples were assessed using DBS (mean, 240.1 ng/mL, *n* = 49) compared with assessments of plasma concentrations (310.1 ng/mL, *n* = 17); however, both methods resulted in similar concentrations at one year (108.4 ng/mL in 49 individuals vs. 117.9 ng/mL in 17 individuals, respectively).

For patients with type 3 GD, chitotriosidase and lyso-GL1 concentrations were shown to remain low and stable with long-term treatment for most patients; however, insufficient data were available to calculate estimated rates of change owing to low patient numbers for those with available data and short duration of treatment.

### 3.8. Skeletal Abnormalities

Centers reported a total of 819 bone abnormalities in 376 patients, 254 (31.0%) of which were bone marrow infiltration, 108 (13.2%) were Erlenmeyer flask deformities, 58 (7.1%) were avascular necrosis, and 7 (0.9%) were fractures (see Table 4 for differences between patients <18 years and patients ≥18 years).

For patients with type 3 GD, a total of 14 bone abnormalities were reported in 20 patients, 3 (21.4) of which were bone marrow infiltration, 3 (21.4) were Erlenmeyer flask deformities, and 8 (57.1) were not specified. All of these bone abnormalities occurred in patients <18 years of age.

### 3.9. Summary of Adverse Events

A total of 1100 AEs were reported by 129 (34.3%) patients during 19 years of velaglucerase alfa treatment (38.9 AEs per 100 patient years of infusions). AEs were reported by fewer children than adults (27.5% vs. 36.9%, respectively), but by more patients who underwent a total splenectomy than those with intact spleens (53.1% vs. 32.6%, respectively). Overall, 66 AEs in 33 (8.8%) patients were considered related to velaglucerase alfa, none of which were considered serious (Table 5). A total of 54 infusion-related AEs related to treatment were reported by 22 (5.9%) patients. The most common velaglucerase alfa-related AEs were headache (six patients); dizziness (five patients); infusion-related reactions and hypertension (four patients each); and back pain, bone pain, hypertension, and hypotension (three patients each; Table 5). No AEs led to discontinuation of treatment. Few velaglucerase alfa-related AEs were reported in patients <18 years of age, with headache, hypotension, and tachycardia occurring in two patients each.

There were 21 deaths overall, 16 of whom were aged >60 years at time of death. None were considered related to treatment. Causes of death were predominantly attributed to cancer (10 patients) and cardiac events (four patients). AEs recorded at the time of death, though not necessarily attributed as causes of death, included respiratory and other causes (headache, head injury, hepatomegaly, suicide, pneumonia, renal failure, respiratory tract infection, seizures, progression of GD). AEs at the time of death were not provided for one patient.

## 4. Discussion

In this analysis of data from the GOS registry, long-term improvements in hematologic and visceral parameters and biomarker concentrations were observed in a cohort of 376 patients with type 1 or type 3 GD treated only with velaglucerase alfa. Consistent with findings from clinical trials and registry data [7,8,11], rapid responses in hematologic and visceral parameters were observed within one year of velaglucerase alfa treatment initiation for most patients with baseline values outside normal thresholds, with those with baseline values indicating severe disease showing the greatest improvements over time. Improvements were maintained long-term for up to 19 years of follow-up.

Few patients overall had baseline platelet counts above the diagnostic threshold for thrombocytopenia. These populations consisted largely of children <18 years of age and those who had undergone total splenectomy. In addition to higher baseline platelet counts, children showed greater initial improvements than adults as well as fewer skeletal abnormalities, which is possibly reflective of treatment initiation occurring earlier in the disease course; despite this, long-term improvements with velaglucerase alfa treatment were similar for both children and adults. Patients who had undergone a total splenectomy had platelet counts above the threshold for thrombocytopenia at all time-points, although none above the threshold for thrombocytosis (>450 × 10^9^/L) [23], in keeping with the role of the spleen in platelet circulation, and in line with previous findings [11].

Consistent with previously published data [10], decreases in liver and spleen volumes were observed with velaglucerase alfa treatment in most patients and were maintained long-term. Nearly two-thirds of patients achieved a liver size < 1.25 MN, the standard diagnostic threshold for hepatomegaly [11,21,22], by one year, and this was maintained over long-term follow-up. However, while a decrease in median spleen size was observed after one year, fewer patients achieved the standard threshold for splenomegaly, <5.0 MN [17], although three-quarters of patients maintained or achieved the therapeutic target for patients with GD of <8.0 MN [17,19]. In GD, increases in spleen volume are often considerably greater than liver volume increases [1], suggestive of a requirement for longer-duration or more aggressive treatment to achieve normal splenomegaly thresholds. In the present analysis, smaller reductions in both liver and spleen volumes over time were observed in children (age < 18 years at baseline) compared with adults, which is possibly an effect of normal childhood growth of liver and spleen volume during the follow-up period.

Decreases in plasma chitotriosidase activity and blood and plasma lyso-GL1 concentrations, observed within a year of treatment initiation and maintained long-term, were in line with improvements in hemoglobin concentrations, platelet counts, and liver and spleen volumes, and consistent with previous findings with velaglucerase alfa [5] and other GD-specific treatments [12,24,25]. Lyso-GL1, a downstream metabolic product of glucosylceramide, has been found to have 100% sensitivity and specificity for GD [26,27], and has been implicated in the pathogenesis of the disease [28]. While chitotriosidase is predominantly used to assess severity of GD and response to treatment, assessment of this biomarker has limitations. Tests for this biomarker are not standardized between laboratories [29], although values below the threshold of ~90 nmol/mL/h are considered ‘normal’ within the scientific literature. Further, more than a third of the general population are estimated to have inherited chitotriosidase deficiency [30], limiting the value of this assay. To account for this, data for patients with chitotriosidase activity < 50 nmol/mL/h at baseline [31,32] were excluded from the analysis of this parameter [30,33].

Few patients with type 3 GD were included in the analyses, likely due to exclusion of patients with this type of GD from most velaglucerase alfa clinical trials, as well as exclusion or delayed inclusion of type 3 GD as a clinical indication for velaglucerase alfa in the US and other countries. Both factors translate into low recruitment into the GOS patient registry. The predominance of centers from the US, Israel, and Europe participating in GOS, which have a high prevalence of type 1 GD, may have further contributed to this imbalance. Nonetheless, hematologic and visceral parameters were found to improve or remain stable over 12 years of follow-up in patients with type 3 GD, consistent with findings for patients with type 1 GD, although spleen volume was found to increase over time in two patients. These findings must be interpreted with caution owing to the small sample size, but appear encouraging for the long-term treatment of non-neurologic manifestations in patients with type 3 GD.

The incidence of AEs was in alignment with previous findings from clinical trials [7,8], with no new long-term emerging safety signals. In this analysis, calculation of the annual incidence of AEs was based on an assumption of linear change over time, which may not be reflective of real-world experience; however, the overall low incidence of AEs and low rate of treatment discontinuation owing to AEs recorded in GOS is consistent with clinical data. Of 21 deaths recorded over the 19-year follow-up period, more than two-thirds occurred in those aged 60 years or older and were predominantly attributed to cancer or cardiac events. The long-term incidence of infusion-related reactions considered related to administration of velaglucerase alfa was higher than observed from phase 3 clinical studies with velaglucerase alfa (5.8% vs. 1%) [34], although with markedly different durations of follow-up (19 vs. 5 years).

There are inherent limitations associated with the collection and analysis of registry data, which should be considered when interpreting the findings of registry studies. Participation in GOS is voluntary and limited to patients under the care of participating centers. A high proportion of patients included in this analysis were from Israel and the US, resulting in an over-representation of patients with Ashkenazi Jewish heritage and of patients with the milder N370S (c.1226A>G; N409S) genotype, while the focus of participating centers across Europe and North and South America resulted in under-representation of patients from Asian and African counties. In addition, dosing patterns and methods for measuring liver and spleen volume and lyso-GL1 concentrations may differ by country, which may impact the ability to generalize these findings to global populations.

As data are collected during routine clinical practice, based on local or regional guidelines and practices, the frequency of visits and the assessments carried out at each visit can vary considerably between patients. The quantity and quality of collected data are dependent on the information entered by participating sites and the willingness of individuals to provide information for entry into the database. As a result, data may be missing or incomplete. Specific skeletal findings were reported here at a lesser frequency than in other studies [35,36]. Owing to data entry by multiple users, data entry may be inconsistent or include errors. Although it is impossible to account for all errors of this type, improbable outliers were excluded from analyses.

In conclusion, these data confirm that long-term treatment with velaglucerase alfa can lead to sustained improvements in hematologic and visceral clinical parameters in patients with type 1 GD, and that long-term stability of systemic symptoms in patients with type 3 GD can be achieved in routine clinical practice. The safety outcomes from GOS provide evidence that velaglucerase alfa treatment exhibits a satisfactory long-term safety profile with up to 19 years of continued treatment.

## Figures and Tables

**Table 1:** Diagnostic threshold and therapeutic targets for GD.

	Standard Diagnostic Range/Threshold	Therapeutic Targets
Hemoglobin concentration, g/L:		≥110 (females and children ≤ 12 years) or ≥120 (males > 12 years) by 1 year, maintained with long-term treatment [17,19]
Children 0–3 years	≥110 [20]	
Children 4–6 years	≥117 [20]	
Children 7–10 years	≥120 [20]	
Children 11–12 years (female)	≥123 [20]	
Children 11–12 years (male)	≥126 [20]	
Females 12–59 years	≥116 [17]	
Females ≥ 59 years	≥115 [17]	
Males 12–59 years	≥127 [17]	
Males ≥ 59 years	≥125 [17]	
Platelet counts, ×10^9^/L	≥150 [18]	1.5- to 2-fold increase by 1 year, ≥100 by 3 years of treatment, maintained with long-term treatment (non-splenectomized patients) Normalization by 1 year of treatment (splenectomized patients) [17,19]
Liver volume, MN	<1.25 [11,21,22]	<1.0 to 1.5 by 1–2 years, depending on baseline liver volume, maintained with long-term treatment [17,19]
Spleen volume, MN	<5 [17]	<2 to 8, depending on baseline spleen volume, maintained with long-term treatment [17,19]

Abbreviations: GD, Gaucher disease; MN, multiples of normal.

**Table 2:** Patient demographics.

	Overall Cohort (*n* = 376)	Type 3 GD Cohort (*n* = 20)
Sex, n (%) Male Female	161 (42.8) 215 (57.2)	4 (20.0) 16 (80.0)
Country, n (%) Argentina Austria Canada France Germany Israel Italy Paraguay Poland Russia Spain UK USA	20 (5.3) 3 (0.8) 10 (2.7) 5 (1.3) 13 (3.5) 152 (40.4) 3 (0.8) 19 (5.1) 3 (0.8) 21 (5.6) 4 (1.1) 38 (10.1) 85 (22.6)	0 0 0 0 0 8 (40.0) 0 0 0 0 0 5 (25.0) 7 (35.0)
Age at symptom onset, years n (missing) Mean (SD) Median (range)	276 (100) 19.1 (17.7) 13.7 (0–85.3)	14 (6) 3.6 (5.8) 1.4 (0–20.0)
Age at diagnosis, years n (missing) Mean (SD) Median (range)	338 (38) 22.0 (18.3) 18.2 (0–85.3)	16 (4) 4.0 (6.2) 1.8 (0–22.0)
Time from symptom onset to diagnosis, years * n (missing) Mean (SD) Median (range)	272 (104) 3.2 (6.4) 0.2 (0–52.4)	13 (7) 1.2 (1.9) 0 (0–6.0)
Time from diagnosis to ERT start, years n (missing) Mean (SD) Median (range)	338 (38) 11.2 (13.3) 5.0 (−1.2 ^†^–74.7)	16 (4) 4.3 (6.5) 0.9 (0–17.1)
Age at velaglucerase alfa initiation, years Mean (SD) Median (range)	33.3 (21.3) 30.9 (0.1–86.1)	7.4 (7.4) 4.0 (0.1–25.0)
Age category at velaglucerase alfa initiation, years, n (%) <18 years ≥18 years	102 (27.1) 274 (72.9)	18 (90.0) 2 (10.0)
Ethnicity, n (%) n (missing) Ashkenazi Jewish Other	301 (75) 137 (45.5) 164 (54.5)	13 (7) 0 13 (100.0)
Spleen status, n (%) Intact Splenectomized (total)	344 (91.5) 32 (8.5)	20 (100.0) 0
GD type, n (%) n (missing) 1 2 3	357 (19) 337 (94.4) 0 20 (5.6)	20 (0) 0 0 20 (100.0)
*GBA1* genotype, n (%) n (missing) N370S/N370S N370S/Other L444P/L444P L444P/other Other	256 (120) 129 (50.4) 89 (34.8) 7 (2.7) 12 (4.7) 19 (7.4)	16 (4) 0 0 7 (43.8) 5 (31.3) 4 (25.0)

Abbreviations: ERT, enzyme replacement therapy; GD, Gaucher disease; SD, standard deviation. * Diagnosis prior to symptom onset owing to family history was set to zero. ^†^ Treatment initiation was prior to diagnosis for 5 patients. Date of diagnosis is not available.

**Table 3:** Estimates for mean annual rate of change in outcomes.

	Overall Cohort			Type 3 GD Cohort		
	*n*	Estimate (SE)	95% CI	*n*	Estimate (SE)	95% CI
Hemoglobin concentration, g/L	309	0.05 (0.01)	0.03, 0.07	17	0.13 (0.05)	0.02, 0.23
Platelet count, ×10^9^/L	311	0.35 (0.04)	0.26, 0.43	18	−0.31 (0.39)	−1.13, 0.52
Liver volume, MN	178	−0.001 (0.0003)	−0.0016, −0.0004	9	−0.005 (0.003)	−0.012, −0.002
Spleen volume, MN	188	−0.03 (0.00)	−0.04, −0.02		ND	ND
Chitotriosidase, nmol/mL/h	210	−135.4 (38,403.1)	−75,974.2, 75,703.4		ND	ND
Lyso-GL1, ng/mL	191	−1.08 (0.20)	−1.48, −0.68		ND	ND

Abbreviations: CI, confidence interval; GD, Gaucher disease; MN, multiples of normal; ND, no data; SE, standard error of the mean.

**Table 4:** Summary of skeletal abnormalities.

Orthopedic Imaging Categories, *n* (%)	Overall Cohort (*n* = 376)	<18 Years (*n* = 102)	≥18 Years (*n* = 274)
Bone marrow infiltration	254 (31.0)	44 (32.8)	210 (30.7)
Erlenmeyer flask deformity	108 (13.2)	15 (11.2)	93 (13.6)
Avascular necrosis	58 (7.1)	1 (0.7)	57 (8.3)
Osteoarthritis	18 (2.2)	0	18 (2.6)
Lytic lesions	11 (1.3)	4 (3.0)	7 (1.0)
Fractures	7 (0.9)	0	7 (1.0)
Missing	3 (0.4)	0	3 (0.4)
Other/not specified	360 (44.0)	70 (52.2)	290 (42.3)
Total	819	134	685

**Table 5:** Summary of adverse events (AEs).

	Overall Cohort (*n* = 376)		<18 Years (*n* = 102)		≥18 Years (*n* = 274)	
	*n* (%)	No. Events	*n* (%)	No. Events	*n* (%)	No. Events
All AEs	129 (34.3)	1100	28 (27.5)	178	101 (36.9)	922
Drug-related AEs *	33 (8.8)	66	7 (6.9)	14	26 (9.5)	52
Infusion-related drug-related AEs *	22 (5.9)	54	4 (3.9)	9	18 (6.6)	45
Serious AEs	79 (21.0)	189	12 (11.8)	18	67 (24.5)	171
Serious drug-related AEs *	0	0	0	0	0	0
AEs leading to death	20 (5.3)	23	2 (2.0)	2	18 (6.6)	21
Most frequent drug-related AEs *						
Headache	6 (1.6)	6	2 (2.0)	2	4 (1.5)	4
Dizziness	5 (1.3)	6	0	0	5 (1.8)	6
Infusion-related reaction	4 (1.1)	4	1 (1.0)	1	3 (1.1)	3
Hypertension	3 (0.8)	4	1 (1.0)	2	2 (0.7)	2
Hypotension	3 (0.8)	3	2 (2.0)	2	1 (0.4)	1
Back pain	3 (0.8)	3	0	0	3 (1.1)	3
Bone pain	3 (0.8)	3	0	0	3 (1.1)	3

* AE with onset within 24 h of velaglucerase alfa dose and considered to be related to treatment, affecting ≥3 patients overall.

## Data Availability

The datasets, including the redacted study protocol, redacted statistical analysis plan, and individual participant data supporting the results of the completed study, will be made available after the publication of final study results within three months from the initial request to researchers who provide a methodologically sound proposal. The data will be provided after its de-identification, in compliance with applicable privacy laws, data protection, and requirements for consent and anonymization.
